# Blood pressure-lowering effect of *Shinrin-yoku* (Forest bathing): a systematic review and meta-analysis

**DOI:** 10.1186/s12906-017-1912-z

**Published:** 2017-08-16

**Authors:** Yuki Ideno, Kunihiko Hayashi, Yukina Abe, Kayo Ueda, Hiroyasu Iso, Mitsuhiko Noda, Jung-Su Lee, Shosuke Suzuki

**Affiliations:** 1 Gunma University Initiative for Advanced Research, 3-39-22 Showa-machi, Maebashi City, Gunma 371-8511 Japan; 2 Graduate School of Health Science, Gunma University, 3-39-22 Showa-machi, Maebashi City, Gunma 371-8511 Japan; 3 Tochigi Prefectural Western District Health and Welfare Services Center, 1664-1 Imamiya-cho, Kanuma City, Tochigi, 322-0068 Japan; 4 Department of Environmental Engineering, Kyoto University Graduate School of Engineering, Yoshida Honmachi, Sakyo-ku, Kyoto, 606-8501 Japan; 5 Public Health, Department of Social and Medicine, Graduate School of Medicine, Osaka University, 1-1 Yamadaoka, Suita, Osaka, 565-0871 Japan; 6 Department of Endocrinology and Diabetes, Saitama Medical University, 38 Morohongo, Moroyama-cho, Iruma-gun, Saitama, 350-0495 Japan; 7 School of Public Health, Graduate School of Medicine, The University of Tokyo, 7-3-1 Hongo, Bunkyo-ku, Tokyo, 113-8654 Japan; 8 Professor Emeritus, Gunma University and NPO International Eco-Health Research Group, 3-39-22 Showa-machi, Maebashi City, Gunma 371-8511 Japan

**Keywords:** Forest bathing, Blood pressure, Pulse rate, Systematic review, Meta-analysis

## Abstract

**Background:**

*Shinrin-yoku* (experiencing the forest atmosphere or forest bathing) has received increasing attention from the perspective of preventive medicine in recent years. Some studies have reported that the forest environment decreases blood pressure. However, little is known about the possibility of anti-hypertensive applications of *Shinrin-yoku*. This study aimed to evaluate preventive or therapeutic effects of the forest environment on blood pressure.

**Methods:**

We systematically reviewed the medical literature and performed a meta-analysis.Four electronic databases were systematically searched for the period before May 2016 with language restriction of English and Japanese. The review considered all published, randomized, controlled trials, cohort studies, and comparative studies that evaluated the effects of the forest environment on changes in systolic blood pressure. A subsequent meta-analysis was performed.

**Results:**

Twenty trials involving 732 participants were reviewed. Systolic blood pressure of the forest environment was significantly lower than that of the non-forest environment. Additionally, diastolic blood pressure of the forest environment was significantly lower than that of the non-forest environment.

**Conclusions:**

This systematic review shows a significant effect of *Shinrin-yoku* on reduction of blood pressure.

**Electronic supplementary material:**

The online version of this article (doi:10.1186/s12906-017-1912-z) contains supplementary material, which is available to authorized users.

## Background

*Shinrin-yoku* (experiencing the forest atmosphere or forest bathing) has received increasing attention from the perspective of preventive medicine in recent years [1–4]. Observational studies have suggested an association of exposure to nature and green vegetation with various health outcomes, including cardiovascular diseases [5]. Some experimental studies have reported the physiological effects of the forest environment (walking in, sitting in, and/or viewing the forest). They reported that the forest environment decreases levels of stress hormones [6–11], blood pressure [6, 12–14], and heart rate (HR) [15, 16], and induce relaxation effects. Changes in urinary adrenaline and noradrenaline levels [6, 7, 9, 10, 12] and HR reflect the autonomic nervous system. Additionally, the autonomic nervous system plays an important role in regulation of blood pressure. Taken together, these findings suggest that the forest environment causes a reduction in blood pressure or prevents hypertension. However, most previous studies did not have a sufficient sample size and their purpose was to examine the reduction of stress or induction of relaxation. Therefore, little is known regarding the possibility of anti-hypertensive applications of *Shinrin-yoku*.

This study aimed to evaluate preventive or therapeutic effects of the forest environment on blood pressure. We systematically reviewed the medical literature and performed a meta-analysis. Further, we examined if there were differences in effects according to the characteristics of participants, such as age, sex, and blood pressure, before intervention.

## Methods

### Selection criteria

This review considered all published, randomized, controlled trials (RCTs), cohort studies, and comparative studies that evaluated the effects of the forest environment on changes in systolic blood pressure (SBP). Participants included adults.

### Outcome measures

#### Primary outcome

The primary outcome was SBP. Provided there were sufficient included trials, sensitivity analyses were performed to explore the influence of the characteristics of participants, such as age, sex, and blood pressure, before intervention on effect size.

#### Secondary outcomes

The secondary outcomes included diastolic blood pressure (DBP) and HR or pulse rate (PR). In this review, we assumed that HR and PR were indices of autonomic nervous activity, and conducted the meta-analysis without distinguishing between them.

### Search strategy

We searched electronic databases (PubMed, Cochrane library, CINAHL, and Japan Medical Abstracts Society Database) from their inception to May 2016 with language restriction of English and Japanese. We also searched the references of relevant studies. The search terms were as follows: (1) forest environment, OR forest area, OR shinrin, OR shinrin-yoku, OR shinrinyoku, OR forest walking, OR forest treatment, OR forest healing, OR forest remedy, OR forest therapy, OR forest basking, OR forest bathing, OR forest viewing, OR phytoncide; (2) blood pressure OR hypertension; (3) random, OR (single-blind method OR double-blind method), OR (single, OR double, OR treble, OR triple), OR (cohort, OR case–control, OR controlled study, OR control group, OR comparative study); and (4) 1, AND 2, AND 3.

### Data collection

Three investigators (YA, YI, SS) extracted the literature, which were retrieved from the medical databases and manual searches. Two of three reviewers (YA, YI, KH) evaluated the full text of the extracted literature independently and discrepancies were resolved by discussion. We extracted information on the following: study characteristics (authors, country, design, year of publication, sample size, duration of intervention, and follow-up); participants’ characteristics (age, sex); intervention (forest exposure and comparators); and outcomes (blood pressure and HR or PR). We extracted the mean change from baseline and standard deviation (SD) and/or before and after mean scores and corresponding SDs.

### Risk of bias

Risk of bias was assessed using Cochrane’s risk of bias tool (random sequence generation, allocation concealment, blinding of participants and personnel, blinding of outcome assessor, incomplete outcome data, selective reporting, other bias).

### Data analysis

Treatment effect size was standardized by mean differences (MDs) that were obtained by dividing changes from baseline (or the difference between before and after treatment) by the pooled SD. Results for the comparative effect are shown by the mean difference estimates and 95% confidence intervals (CIs). We used a standard inverse variance random effects model for meta-analyses. Heterogeneity was assessed by I^2^ statistics. Publication bias or small study effects were checked by the conventional funnel plot. Statistical analyses were performed by Cochrane Review Manager software (RevMan) ver5.1 (The Nordic Cochrane Center, The Cochrane Collaboration). When we used the generic inverse variance method to incorporate cross-over studies into the meta-analysis, we used the formula listed in the Cochrane Handbook for Systematic Reviews of Interventions, section 16.4.6.1 [17], to calculate the SD of the difference between treatment and control. This study was registered as PROSPERO CRD42016038286.

## Results

### Results of the search

**Fig. 1:**
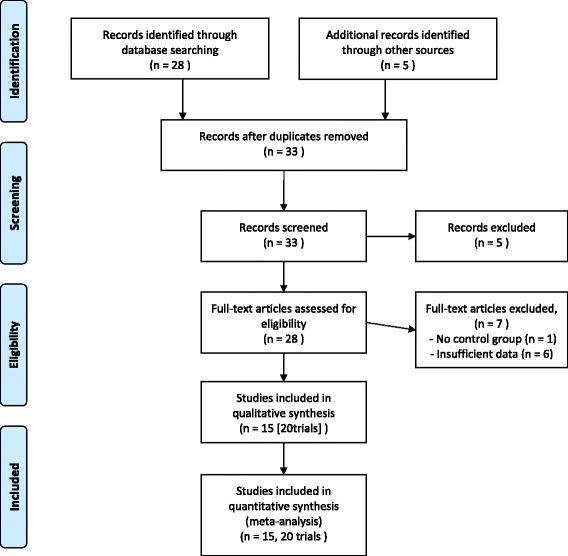
Flow chart of the study selection process for the present study

The initial search identified 33 records, of which 28 full papers were identified for further examination. We excluded five papers on the basis of their abstracts because they did not meet the selection criteria of the study design. After screening the full text of the 28 selected papers, 21 met the inclusion criteria; seven were excluded because of no control group (*n* = 1) or insufficient data (*n* = 6). Figure 1 shows the details of the study selection process. Because these 21 papers included duplicated papers of identical studies, we identified 15 studies, including 12 studies from 12 papers [10, 11, 16, 18–26], two studies from three papers [27–29], and one study from six papers [30–35]. Additionally, two papers reported outcomes according to sex [16, 30], and three papers reported outcomes separately according to two intervention methods [20, 21, 23]. Therefore, we considered them as separate trials, and finally included 20 trials in analyses. To distinguish these separate trials, we added the numbers “-1” and “-2” to the dates of the trials (Additional file 1).

### Study design

A detailed description of the characteristics of included trials is shown in Additional file 1. Of these 20 trials, five were parallel-group comparison studies and 15 were cross-over studies.

### Participants

In total, 732 participants were included in the 20 trials. Sample sizes ranged from 6 to 268, and the median size was 12 participants. The mean age of participants in included trials ranged from 18 to 80 years. Participants of 12 trials were only males, four trials included only females, and four trials did not distinguish sex.

### Interventions

All trials used the forest environment. The main intervention methods were walking in forest areas (11 trials), and sitting and viewing forest landscapes (7 trials). These intervention methods were compared with the same activities in non-forest environment-like city areas (13 trials), with sitting in a room (4 trials), or with only measuring blood pressure in daily life (2 trials). The duration of interventions in most trials was within 2 h (16 trials), but the duration of two trials was longer than 1 day.

### Outcomes

Of the 20 trials that reported values of SBP, 17 reported values of DBP, five reported values of HR, and eight reported values of PR.

### Countries

Trials were conducted in three countries, with 17 trials conducted in Japan, two in Korea, and one in China.

### Systolic blood pressure

**Fig. 2:**
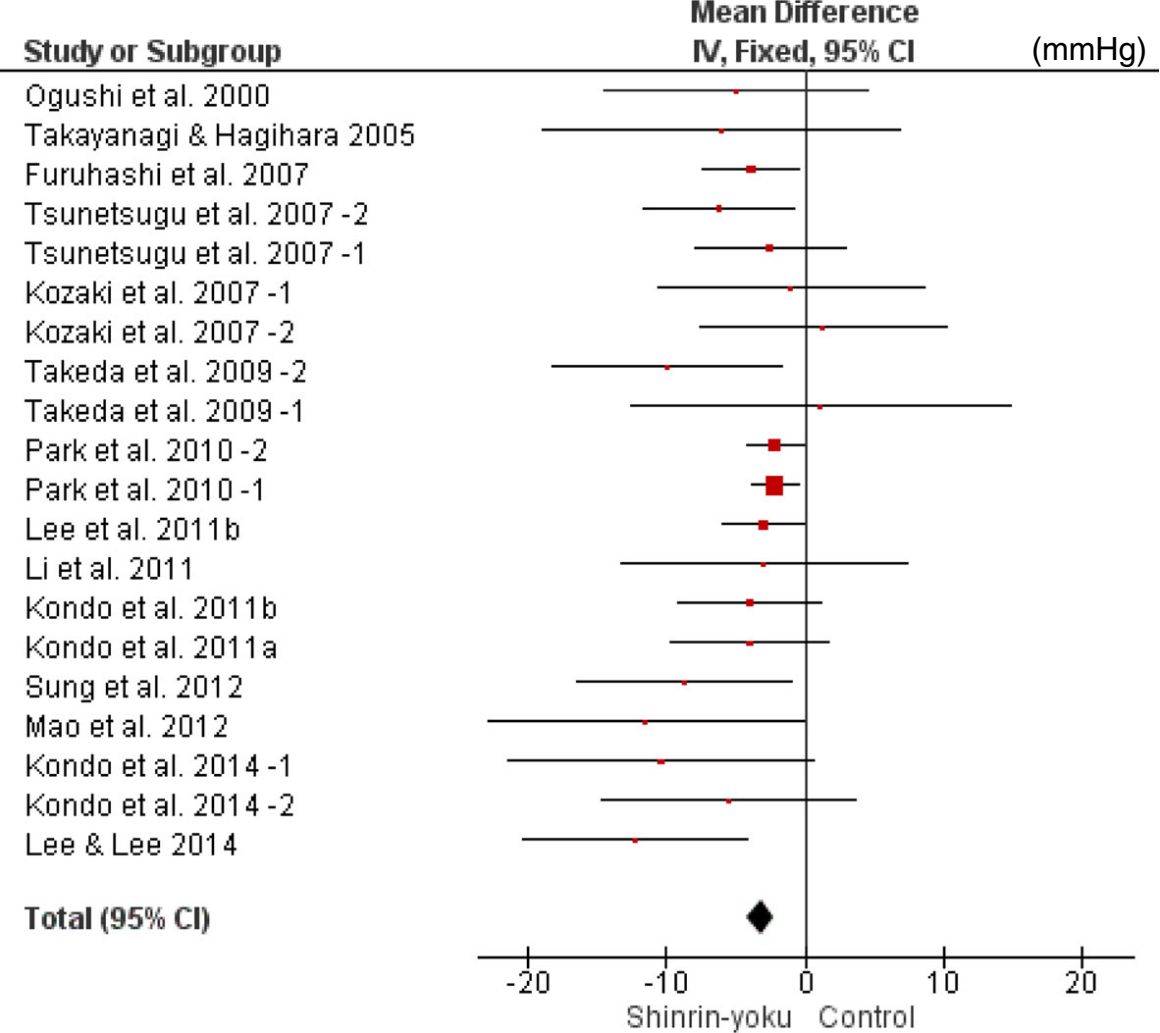
Forest plot comparing changes in SBP (mmHg) between the forest environment and the non-forest environment based on 20 studies or subgroups CI, confidence interval.

**Fig. 3:**
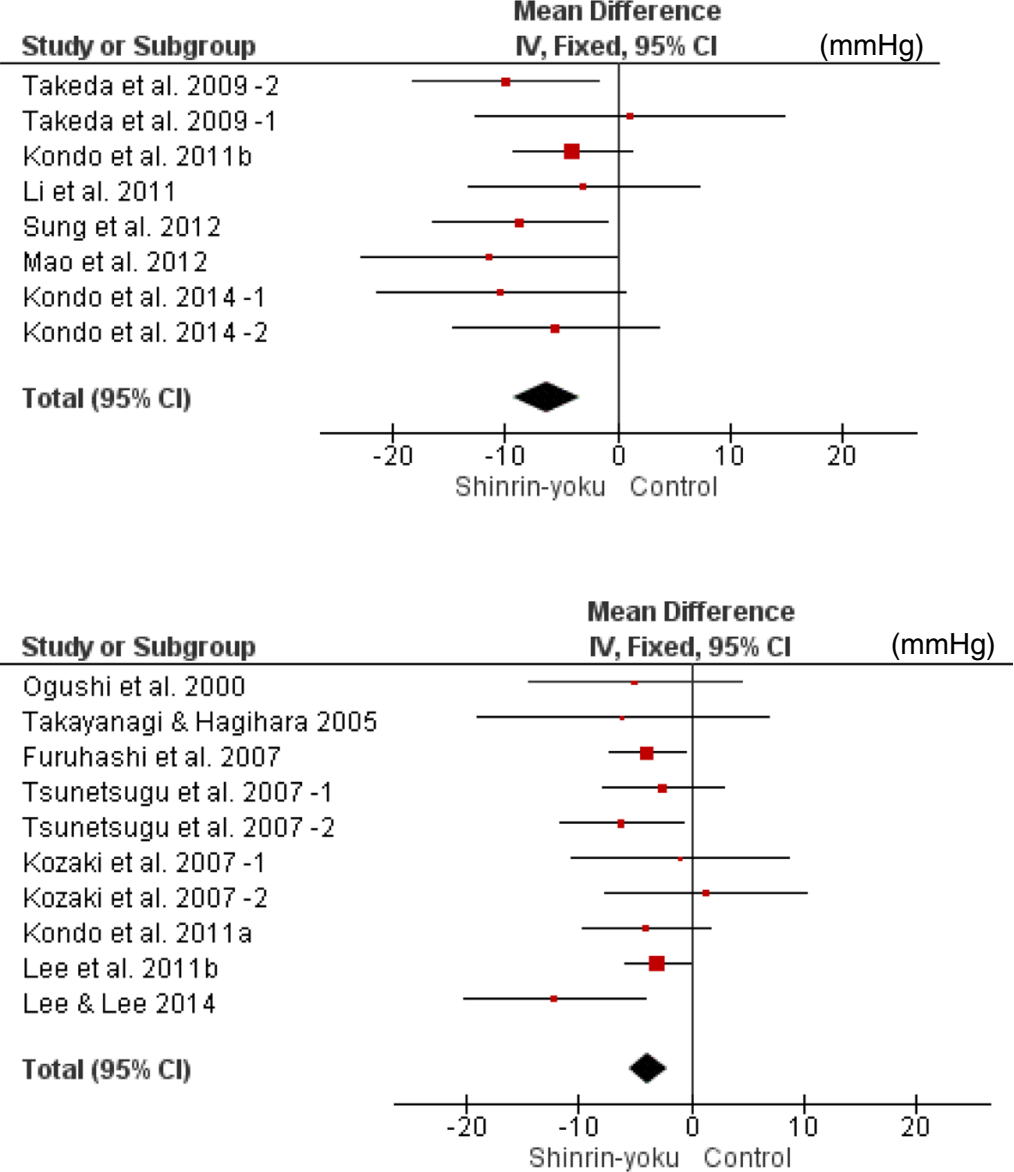
Two forest plots of the change in SBP (mmHg) in the two subgroup analyses of SBP levels of 1) ≥ 130 and 2) < 130 mmHg before the intervention. 1) SBP ≥ 130 mmHg. 2) SBP < 130 mmHg. CI, confidence interval

The meta-analysis was conducted using all 20 trials. SBP of the forest environment was significantly lower than that of the non-forest environment (MD −3.15 mmHg; 95% CI −4.12 to −2.18; *P* < 0.001; I^2^ = 1%; 732 participants; 20 trials) (Table 1 **,** Fig. 2). They were divided into the SBP ≥ 130 mmHg group and the SBP < 130 mmHg group to investigate heterogeneous effects in hypertensive and normotensive subjects, and meta-analyses were conducted. In both groups, SBP of the forest environment was significantly lower than that in the non-forest environment (SBP ≥ 130 mmHg: MD −6.33 mmHg; 95% CI −9.35 to −3.31; *P* < 0.001; I^2^ = 0%; 136 participants; 8 trials, SBP < 130 mmHg: MD −3.85 mmHg; 95% CI −5.53 to −2.17; *P* < 0.001; I^2^ = 0%; 253 participants; 10 trials) (Table 2 **,** Fig. 3).

**Table 1:** Result of the meta-analysis comparing changes in SBP (mmHg) between the forest environment and the non-forest environment based on 20 studies or subgroups

Study or Subgroup	Sinrin-yoku Total	Control Total	Weight (%)	Mean Difference IV, Fixed	95% CI
Ogushi et al., 2000 [18]	37	20	1.0	−5.00	−14.58 - 4.58
Takayanagi & Hagihara, 2005 [19]	12	12	0.6	−6.10	−19.02 - 6.82
Tsunetsugu et al., 2007–2 [20]	11	*	3.2	−6.20	−11.65 - -0.75
Tsunetsugu et al., 2007–1 [20, 40]	9	*	3.3	−2.50	−7.87 - 2.87
Furuhashi et al., 2007 [22]	12	*	7.8	−3.90	−7.37 - -0.43
Kozaki et al., 2007–1 [21]	11	*	1.0	−1.00	−10.62 - 8.62
Kozaki et al., 2007–2 [21]	11	*	1.2	−1.30	−7.60 - 10.20
Takeda et al., 2009–2 [30–35]	8	*	1.3	−10.00	−18.35 - -1.65
Takeda et al., 2009–1 [30–35]	11	*	0.5	1.10	−12.64 - 14.84
Park et al., 2010–2 [23]	75	*	20.9	−2.16	−4.28 - -0.04
Park et al., 2010–1 [23]	268	*	35.4	−2.16	−3.79 - -0.53
Li et al., 2011 [10]	16	*	0.9	−3.00	−13.33 - 7.33
Lee et al., 2011 [24]	48	*	11.0	−3.00	−5.92 - -0.08
Kondo et al., 2011a [27, 29]	8	*	2.9	−4.00	−9.66 - 1.66
Kondo et al., 2011b [28, 29]	8	*	3.4	−4.00	−9.23 - 1.23
Sung et al., 2012 [25]	28	28	1.5	−8.70	−16.48 - -0.92
Mao et al., 2012 [26]	12	12	0.7	−11.50	−22.87 - -0.13
Kondo et al., 2014–1 [16]	7	*	0.8	−10.40	−21.43 - 0.63
Kondo et al., 2014–2 [16]	6	*	1.1	−5.50	−14.73 - 3.73
Lee & Lee, 2014 [11]	43	19	1.4	−12.25	−20.36 - -4.14
Total	641	91**	100.0	−3.15	−4.12 - -2.18

CI, confidence interval; df, degrees of freedom

*Cross-over design (same number as in Shinrin-yoku)

**Total number excluding the cross-over design

**Table 2:** Results of the meta-analysis of the change in SBP (mmHg) in the two subgroup analyses of SBP levels of 1) ≥ 130 and 2) < 130 mmHg before the intervention

Study or Subgroup	Sinrin-yoku Total	Control Total	Weight (%)	Mean Difference IV, Fixed	95% CI
1) SBP ≥ 130 mmHg					
Tsunetsugu et al., 2007–2 [20]	8	*	13.1	−10.00	−18.35 - -1.65
Tsunetsugu et al., 2007–1 [20, 40]	11	*	4.8	1.10	−12.64 - 14.84
Kondo et al., 2011b [28, 29]	8	*	33.3	−4.00	−9.23 - 1.23
Li et al., 2011 [10]	16	*	8.5	−3.00	−13.33 - 7.33
Sung et al., 2012 [25]	28	28	15.1	−8.70	−16.48 - -0.92
Mao et al., 2012 [25]	12	12	7.1	−11.50	−22.87 - -0.13
Kondo et al., 2014–1 [16]	7	*	7.5	−10.40	−21.43 - 0.63
Kondo et al., 2014–2 [16]	6	*	10.7	−5.50	−14.73 - 3.73
2) SBP < 130 mmHg					
Ogushi et al., 2000 [18]	37	20	3.1	−5.00	−14.58 - 4.58
Takayanagi & Hagihara, 2005 [19]	12	12	1.7	−6.10	−19.02 - 6.82
Furuhashi et al., 2007 [22]	12	*	23.4	−3.90	−7.37 - -0.43
Tsunetsugu et al., 2007–1 [20, 40]	9	*	9.8	−2.50	−7.87 - 2.87
Tsunetsugu et al., 2007–2 [20]	11	*	9.5	−6.20	−11.65 - -0.75
Kozaki et al., 2007–1 [21]	11	*	3.0	−1.00	−10.62 - 8.62
Kozaki et al., 2007–2 [21]	11	*	3.6	−1.30	−7.60 - 10.20
Kondo et al., 2011a [27, 29]	8	*	8.8	−4.00	−9.66 - 1.66
Lee et al., 2011 [24]	48	*	33.0	−3.00	−5.92 - -0.08
Lee & Lee, 2014 [11]	43	19	4.3	−12.25	−20.36 - -4.14
Total	202	51**	100.0	−3.85	−5.53 - -2.17

Heterogeneity: Chi^2^ = 4.73, df = 7 (*P* = 0.69); I^2^ = 0%

Test for overall effect: Z = 4.11 (*P* < 0.0001)

Heterogeneity: Chi^2^ = 7.20, df = 9 (*P* = 0.62); I^2^ = 0%

Test for overall effect: Z = 4.50 (*P* < 0.00001)

CI, confidence interval; df, degrees of freedom

*Cross-over design (same number as in *Shinrin-yoku*)

**Total number excluding the cross-over design

**Fig. 4:**
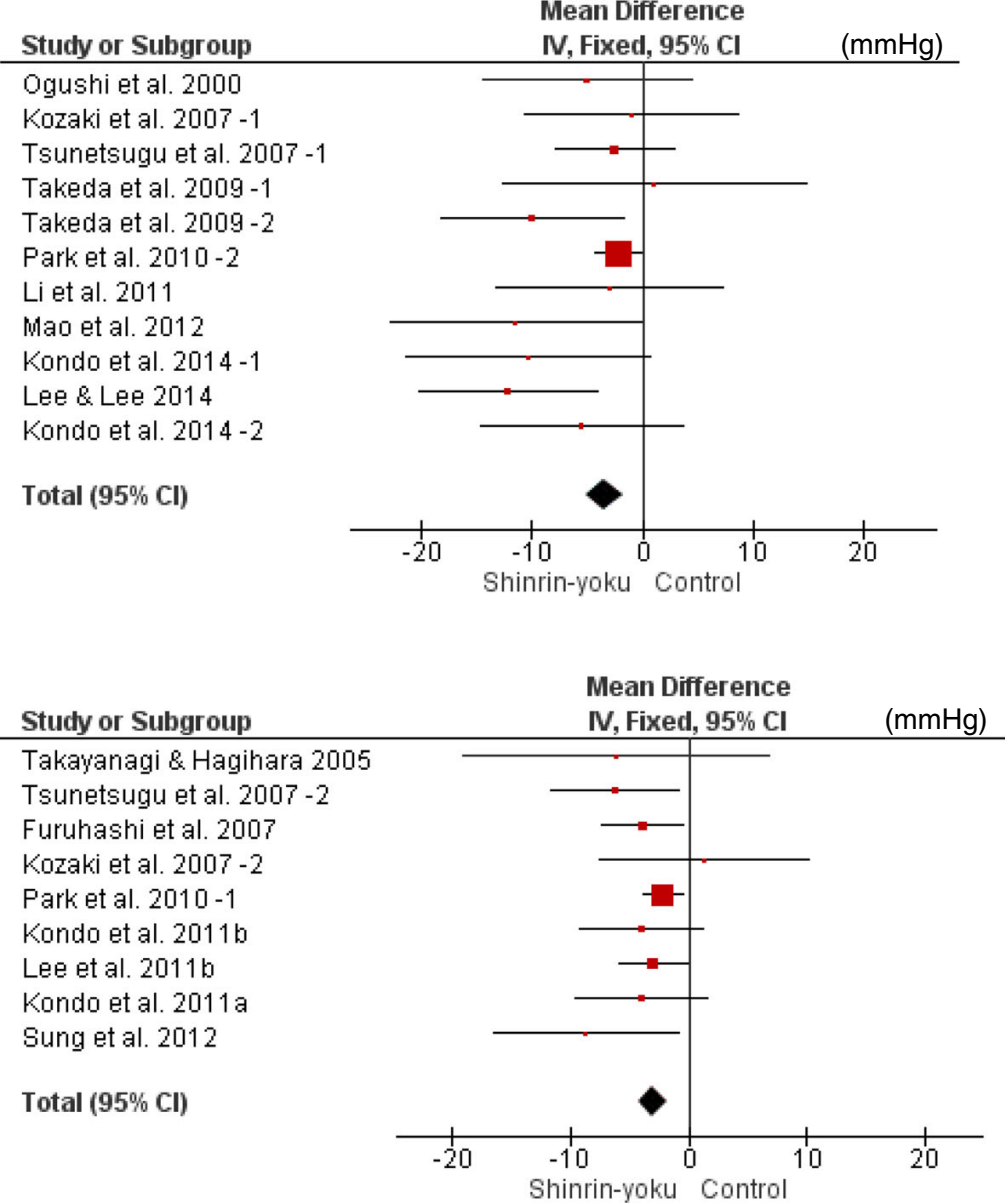
Two forest plots of the change in SBP (mmHg) in the two subgroup analyses of differences in intervention methods (walking and non-walking). 1) Walking group. 2) Non-walking group. CI, confidence interval

**Fig. 5:**
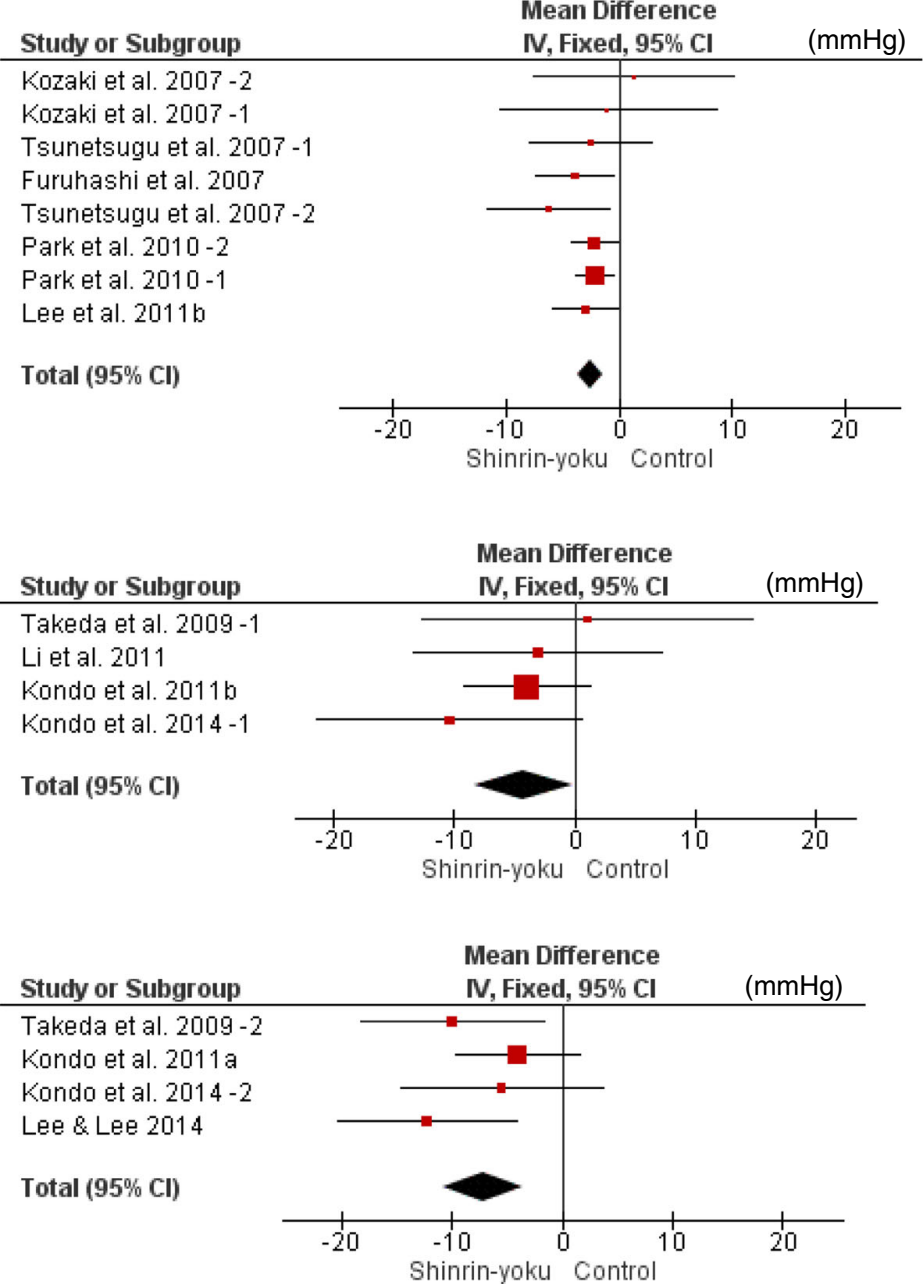
Three forest plots of the change in SBP (mmHg) in the two subgroup analyses of the three groups of sex and age differences. 1) Young male (university student) group. 2) Middle-aged or older male group. 3) Middle-aged or older female group. CI, confidence interval

We then divided these trials into the walking group and the non-walking group to examine the difference in intervention methods, and conducted meta-analyses. In both groups, SBP of the forest environment was lower than that of the non-forest environment (walking: MD −3.48 mmHg; 95% CI −5.17 to −1.80; *P* < 0.001; I^2^ = 22%; 286 participants; 11 trials, non-walking: MD −2.99 mmHg; 95% CI −4.17 to −1.80; *P* < 0.001; I^2^ = 0%; 446 participants; 9 trials) (Table 3 **,** Fig. 4). Additionally, the trials were divided into the young male group, the middle-aged or older male group, and the middle-aged or older female group to examine differences in age and sex. Meta-analyses were then conducted. In all of the groups, SBP of the forest environment was lower than that of the non-forest environment (young males: MD −2.53 mmHg; 95% CI −3.59 to −1.48; *P* < 0.001; I^2^ = 0%; 445 participants; 8 trials, middle-aged or older males: MD −4.27 mmHg; 95% CI −8.38 to −0.17; *P* = 0.04; I^2^ = 0%; 42 participants; 4 trials, middle-aged or older females: MD −7.16 mmHg; 95% CI −10.88 to −3.45; *P* < 0.001; I^2^ = 8%; 84 participants; 4 trials) (Table 4 **,** Fig. 5).

**Table 3:** Results of the meta-analysis of the change in SBP (mmHg) in the two subgroup analyses of differences in intervention methods (walking and non-walking)

Study or Subgroup	Sinrin-yoku Total	Control Total	Weight (%)	Mean Difference IV, Fixed	95% CI
1) Walking group					
Ogushi et al., 2000 [18]	37	20	3.1	−5.00	−14.58 - 4.58
Kozaki et al., 2007–1 [21]	11	*	3.1	−1.00	−10.62 - 8.62
Tsunetsugu et al., 2007–1 [20, 40]	9	*	9.9	−2.50	−7.87 - 2.87
Takeda et al., 2009–1 [30–35]	11	*	1.5	1.10	−12.64 - 14.84
Takeda et al., 2009–2 [30–35]	8	*	4.1	−10.00	−18.35 - -1.65
Park et al., 2010–2 [23]	75	*	63.5	−2.16	−4.28 - -0.04
Li et al., 2011 [10]	16	*	2.7	−3.00	−13.33 - 7.33
Mao et al., 2012 [26]	12	12	2.2	−11.50	−22.87 - -0.13
Kondo et al., 2014–1 [16]	7	*	2.3	−10.40	−21.43 - 0.63
Lee & Lee, 2014 [11]	43	19	4.3	−12.25	−20.36 - -4.14
Kondo et al., 2014–2 [16]	6	*	3.3	−5.50	−14.73 - 3.73
Total	235	51**	100.0	−3.48	−5.17 - -1.80
2) Non-walking group					
Takayanagi & Hagihara, 2005 [19]	12	12	0.8	−6.10	−19.02 - 6.82
Kozaki et al., 2007–2 [21]	11	*	1.8	−1.30	−7.60 - 10.20
Tsunetsugu et al., 2007–2 [20]	11	*	4.7	−6.20	−11.65 - -0.75
Furuhashi et al., 2007 [22]	12	*	11.6	−3.90	−7.37 - -0.43
Park et al., 2010–1 [23]	268	*	52.9	−2.16	−3.79 - -0.53
Kondo et al., 2011b [27–29]	8	*	5.1	−4.00	−9.23 - 1.23
Lee et al., 2011 [24]	48	*	16.4	−3.00	−5.92 - -0.08
Kondo et al., 2011a [27, 29]	8	*	4.4	−4.00	−9.66 - 1.66
Sung et al., 2012 [25]	28	28	2.3	−8.70	−16.48 - -0.92
Total	406	40**	100.0	−2.99	−4.17 - -1.80

Heterogeneity: Chi^2^ = 12.85, df = 10 (*P* = 0.23); I^2^ = 22%

Test for overall effect: Z = 4.05 (*P* < 0.0001)

Heterogeneity: Chi^2^ = 6.05, df = 8 (*P* = 0.64); I^2^ = 0%

Test for overall effect: Z = 4.95 (*P* < 0.00001)

CI, confidence interval; df, degrees of freedom

*Cross-over design (same number as in *Shinrin-yoku*)

**Total number excluding the cross-over design

**Table 4:** Results of the meta-analysis of the change in SBP (mmHg) in the two subgroup analyses of the three groups of sex and age differences

Study or Subgroup	Sinrin-yoku Total	Control Total	Weight (%)	Mean Difference IV, Fixed	95% CI
1) Young male (university student) group					
Kozaki et al., 2007–2 [21]	11	*	1.4	−1.30	−7.60 - 10.20
Kozaki et al., 2007–1 [21]	11	*	1.2	−1.00	−10.62 - 8.62
Tsunetsugu et al., 2007–1 [20, 40]	9	*	3.9	−2.50	−7.87 - 2.87
Furuhashi et al., 2007 [22]	12	*	9.3	−3.90	−7.37 - -0.43
Tsunetsugu et al., 2007–2 [20]	11	*	3.8	−6.20	−11.65 - -0.75
Park et al., 2010–2 [23]	75	*	25.0	−2.16	−4.28 - -0.04
Park et al., 2010–1 [23]	268	*	42.3	−2.16	−3.79 - -0.53
Lee et al., 2011 [24]	48	*	13.1	−3.00	−5.92 - -0.08
Total	445	0**	100.0	−2.53	−3.59 - -1.48
2) Middle-aged or older male group					
Takeda et al., 2009–1 [30–35]	11	*	8.9	1.10	−12.64 - 14.84
Li et al., 2011 [10]	16	*	15.8	−3.00	−13.33 - 7.33
Kondo et al., 2011b [28, 29]	8	*	61.5	−4.00	−9.23 - 1.23
Kondo et al., 2014–1 [16]	7	*	13.8	−10.40	−21.43 - 0.63
Total	42	0**	100.0	−4.27	−8.38 - -0.17
3) Middle-aged or older female group					
Takeda et al., 2009–2 [30–35]	8	*	19.8	−10.00	−18.35 - -1.65
Kondo et al., 2011a [27, 29]	8	*	43.0	−4.00	−9.66 - 1.66
Kondo et al., 2014–2 [16]	6	*	16.2	−5.50	−14.73 - 3.73
Lee & Lee, 2014 [11]	43	19	21.0	−12.25	−20.36 - -4.14
Total	65	19**	100.0	−7.16	−10.88 - -3.45

Heterogeneity: Chi^2^ = 3.57, df = 7 (*P* = 0.83); I^2^ = 0%

Test for overall effect: Z = 4.70 (*P* < 0.00001)

Heterogeneity: Chi^2^ = 3.28, df = 3 (*P* = 0.35); I^2^ = 8%

Test for overall effect: Z = 3.78 (*P* < 0.0002)

CI, confidence interval; df, degrees of freedom

*Cross-over design (same number as in *Shinrin-yok*u)

**Total number excluding the cross-over design

### Diastolic blood pressure

Seventeen trials were suitable for meta-analysis. DBP of the forest environment was significantly lower than that of the non-forest environment (MD −1.75 mmHg; 95% CI −2.38 to −1.13; *P* < 0.001; I^2^ = 24%; 705 participants; 17 trials).

### Heart rate and pulse rate

Five trials reported values of HR and eight trials reported values of PR. We conducted meta-analysis using these 13 trials. HR and PR of the forest environment were significantly lower than those of the non-forest environment (MD −3.84 bpm; 95% CI −5.27 to −2.40; *P* < 0.001; I^2^ = 39%; 563 participants; 13 trials).

### Publication bias

**Fig. 6:**
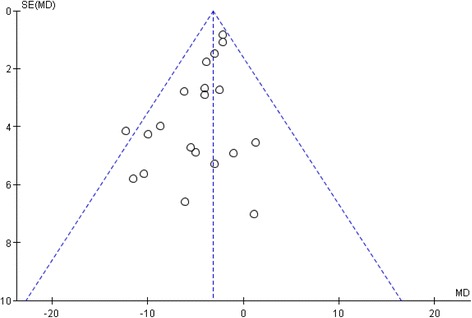
Funnel plot, using data from 20 trials of therapeutic effects of the forest environment on systolic blood pressure. SE, standard error; MD, mean difference

The funnel plot demonstrated that toward the bottom of the graph most studies appear toward the left (indicating more risk), which is consistent with the possibility that some studies are missing from the right (Fig. 6). But, this plot showed an almost symmetrical shape. It suggested weak evidence of publication bias.

## Discussion

This systematic review examined the evidence for effects of the forest environment on blood pressure. The review identified 20 trials (15 studies), involving 732 participants. The majority of trials were cross-over studies (15 trials), and only five trials were parallel-group comparison studies, which had a separate control group from the experimental group. The results of meta-analysis showed that SBP and DBP of the forest environment were significantly lower than those of the non-forest environment. Taken together, these findings suggest that the forest environment may have anti-hypertensive effects.

For analysis according to participants’ characteristics, we conducted sub-group analyses considering SBP levels before intervention and age. The trials were divided into the high SBP group (≥ 130 mmHg) and the low SBP group (< 130 mmHg) according to the value of SBP before intervention. Meta-analysis showed that SBP of the forest environment was significantly lower than that of the non-forest environment. When we compared the MD of both groups, the MD in the high SBP group was lower than that in the low SBP group. This finding suggests that the forest environment may have a larger effect on lowering SBP in hypertensive people than in normotensive people.

We then divided subjects into the young male group, the middle-aged or older male group, and the middle-aged or older female group. Although all of the groups showed that SBP of the forest environment was lower than that of the non-forest environment, the MD of the older groups was lower than that of the younger group. This finding suggests that the forest environment has a larger effect on lowering SBP in older people than in younger people. However, all trials for male university students comprised the low SBP group and blood pressure rises with age. Therefore, rather than being affected by age, the forest environment might have a larger effect on lowering SBP in people with higher blood pressure than in those with lower blood pressure.

An observational study showed a positive association of exposure to the forest environment with reduced blood pressure in children [36]. However, the mechanism of how the forest environment reduces blood pressure is unclear. The present systematic review showed that HR and PR, which were evaluated as indices of autonomic nervous activity, were significantly decreased after the interventions. This finding indicates that the forest environment reduces sympathetic nerve activity and increases parasympathetic nerve activity. Additionally, some studies have reported that the forest environment significantly reduces urinary adrenaline and noradrenaline levels [6, 7, 9, 10, 12]. Adrenaline and noradrenaline levels are indices of autonomic nervous activity, and their reduction suggests that sympathetic nerve activity is reduced. This review suggests that the autonomic nervous system plays an important role in the regulation of blood pressure.

We also divided the trials into the walking group and the non-walking group to examine differences in intervention methods, and conducted sub-group meta-analyses. In both groups, SBP of the forest environment was significantly lower than that in the non-forest environment. There was not a large difference in the MD between these groups. This finding suggests that the effect of the forest environment on blood pressure is not due to physical activity. Some studies have shown that phytoncides (wood essential oil) may have a beneficial effect on blood pressure [10, 37]. Further study is necessary because some factors in the forest environment reduce blood pressure.

This study has several limitations. First, because most trials investigated only immediate effects of the forest environment, we could not evaluate long-term effects. There were only two trials in which the intervention or observation period was longer than 1 day [25, 26], of which Sung et al. [25] used 3 days and Mao et al. [26] used 7 days. Both trials showed that SBP of the forest environment was significantly lower than that of the non-forest environment. However, Sung et al. [25] also reported that SBP at 8 weeks after a 3-day forest therapy program was not significantly different between the forest therapy group and the control group. These findings suggest that repeated exposure to the forest environment may be needed to obtain long-term benefits. Second, we could not characterize individual differences in responses. Although there are individual differences in effects of natural environments, research methods have not been clearly established. Song et al. [38] described that individual differences in responses to forest environments can be explained by type A and Type B behavior patterns. Additionally, Wilder formulated the “law of initial value”, which states that “the higher this ‘initial level’, the smaller is the response to function-raising, the greater is the response to function-depressing agents” [39]. Some studies have reported a significant negative correlation between baseline and the amount of change in the forest environment, such as salivary cortisol concentrations [40] and blood pressure [41]. However, because 15 of 20 trials that were included in this review were cross-over studies, considering individual differences is not so important. Third, we did not consider antihypertensive medication in this review. Further study taking this medication into account is required**.**

The second term of the National Health Promotion Movement in the twenty-first century (Health Japan 21 [the second term]) estimates that lowering average systolic blood pressure by 4 mmHg will lead to a reduction in mortality rate of cerebrovascular disease (males, 8.9%; females, 5.8) and in mortality rate of ischemic heart disease (males, 5.4%; females, 7.2), among 40- to 89-year-old people whose prevalence of high blood pressure is high [42, 43]. Our study showed that the forest environment reduced SBP by 6.33 mmHg in the high SBP group, by 4.27 mmHg in the middle-aged or older male group, and by 7.16 mmHg in the middle-aged and older female group. Therefore, we believe that the forest environment can have anti-hypertensive effects and contribute to health promotion for middle-aged or older people.

## Conclusions

This systematic review shows significant effects of the forest environments on a reduction in blood pressure. In particular, the forest environment has a larger effect on lowering SBP in people with high blood pressure and in middle-aged or older people compared with the non-forest environment.

## Additional file

Additional file 1: Characteristics of included studies. (XLSX 16 kb)
